# A rare finding on computed tomography angiography performed to exclude pulmonary embolism

**DOI:** 10.1007/s12471-022-01743-2

**Published:** 2022-12-12

**Authors:** Vivan J. M. Baggen, Léon J. P. M. van Woerkens, Robert M. Kauling, Atilla Dirkali

**Affiliations:** 1 Albert Schweitzer Ziekenhuis, Dordrecht, The Netherlands; 2 Erasmus Medical Centre, Rotterdam, The Netherlands

A 57-year-old man was evaluated by the pulmonologist because of dyspnoea. His past medical history included hypertension, hypercholesterolaemia, and an evaluation in 2018 because of syncope after intensive sports training. Transthoracic echocardiography at the time showed mild left ventricular hypertrophy and an anomaly which was noticed only in retrospect (Fig. 1a, asterisk). Holter monitoring and exercise electrocardiography were normal. In 2021 the patient was re-evaluated because of reduced exercise tolerance and palpitations. Holter monitoring showed frequent ventricular ectopic beats and some runs of non-sustained ventricular tachycardia (maximum 5 beats). Cardiac catheterisation showed diffuse coronary artery disease with multiple intermediate stenoses, all assessed as non-significant by fractional flow reserve. Treatment with aspirin, statin and beta-blocker was initiated.

**Fig. 1:**
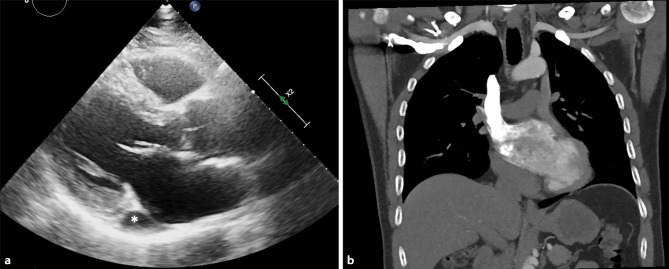
**a** Transthoracic echocardiography, parasternal long-axis view. The *asterisk* indicates an anomaly. **b** Computed tomography angiography performed to rule out pulmonary embolism. Contrast was administered via the right arm, coronal plane

The pulmonologist performed computed tomography angiography to exclude pulmonary embolism. The contrast was administered via the right arm. However, in Fig. 1b no contrast can be seen in the pulmonary artery.

What is your diagnosis: which two congenital anomalies can be seen in Fig. 1?

## Answer

You will find the answer elsewhere in this issue.

